# Association between Oropharyngeal Dysphagia and Malnutrition in Dutch Nursing Home Residents: Results of the National Prevalence Measurement of Quality of Care

**DOI:** 10.1007/s12603-018-1103-8

**Published:** 2018-09-27

**Authors:** Viviënne A. L. Huppertz, R. J. G. Halfens, A. van Helvoort, L. C. P. G. M. de Groot, L. W. J. Baijens, J. M. G. A. Schols

**Affiliations:** 1 Maastricht University, Nutrition and Translational Research in Metabolism (School NUTRIM), Dept. Pulmonology, Maastricht, The Netherlands; 2 Maastricht University, Care and Public Health Research Institute (School CAPHRI), Dept. Health Services Research, Maastricht, The Netherlands; 3 Danone Nutricia Research, Nutricia Advanced Medical Nutrition, Utrecht, The Netherlands; 4 Wageningen University and Research Center, Division of Human Nutrition and Health, Wageningen, The Netherlands; 5 Maastricht University Medical Center, Dept. of Otorhinolaryngology, Head and Neck Surgery, Maastricht, The Netherlands; 6 Maastricht University Medical Center, School for Oncology and Developmental Biology – GROW, Maastricht, The Netherlands; 7 Maastricht University: Department of Pulmonology, P.O. Box 616, 6200 MD Maastricht, The Netherlands

**Keywords:** Oropharyngeal dysphagia, malnutrition, nursing homes

## Abstract

**Objectives:**

Nursing home residents often suffer from multi-morbidities and geriatric syndromes leading to lower quality of life or mortality. Oropharyngeal dysphagia (OD) and malnutrition are profound conditions in this complex profile of multi-morbidities and are associated with deprived mental –and physical health status, e.g. aspiration pneumonia or dehydration. This study aimed to assess the association between OD and malnutrition in Dutch nursing home residents.

**Design:**

Data for this cross-sectional study were obtained from the annual National Prevalence Measurement of Quality of Care (LPZ).

**Setting:**

The National Prevalence Measurement of Quality of Care was conducted in Nursing Homes in The Netherlands.

**Participants:**

Participants were nursing home residents age 65 or older and admitted to psychogeriatric- or somatic wards.

**Measurements:**

The measurements were taken by trained nurses from the participating nursing homes. Anthropometric measurements and unintended weight loss (%) were assessed to determine nutritional status (malnutrition). OD was assessed by means of a standardized questionnaire assessing clinically relevant symptoms of OD such as swallowing problems or sneezing/coughing while swallowing. Cox regression was applied to assess the association between malnutrition and clinically relevant symptoms of OD in older Dutch nursing home residents.

**Results:**

Approximately 12% of the residents suffered from swallowing problems and 7% sneezed/coughed while swallowing liquids or solid foods. Approximately 10% of the residents was malnourished. Residents with OD symptoms were more often malnourished compared to residents without OD symptoms. Approximately 17% of the problematic swallowers were concurrently malnourished. Increased risk for malnutrition was found in residents suffering from swallowing problems (PR 1.5, 95%CI 1.2–1.9), as well as in residents that sneezed/ coughed while swallowing (PR 1.3, 95%CI 1.0–1.7). Stratification based on wards revealed that problematic swallowers from somatic wards were at a high risk of malnutrition (PR 1.9, 95%CI 1.3–2.8).

**Conclusion:**

Clinically relevant symptoms of oropharyngeal dysphagia, such as swallowing problems and sneezing/coughing while swallowing are associated with increased risk of malnutrition in psychogeriatric and somatic Dutch nursing home residents.

## Introduction

Oropharyngeal dysphagia (OD) and malnutrition are conditions that result in lower quality of life and that place people at high risk for co-morbidities and mortality. OD is considered a new geriatric syndrome (1), and frequently occurs in nursing home residents (2, 3), especially in residents who suffer from stroke (4), dementia (5), or from other illnesses or treatments that affect the swallowing mechanism (6). Furthermore, aging related changes in motor- or sensory functions and muscle strength of the oral cavity are shown to affect swallowing capacity and the nutritional status (7, 8). The integrity of functional swallowing capacity is not only of great importance for safe oral intake of nutrition, but also for a safe oral intake of medication in this multi-morbid population.

OD and malnutrition complicate care in older nursing home residents in view of associated health complications, co-morbidities and a deprived mental health status. When OD and malnutrition are underestimated, unrecognized (so-called silent dysphagia (9, 10)) or left untreated, they may lead to aspiration pneumonia or dehydration respectively (11–14) or to feelings of social isolation (15), anxiety or even depression (16). Impaired eating behaviour could also be a consequence of dementia or depression (17, 18) and swallowing capacity or nutritional status may be influenced by side effects of certain antipsychotic drugs (19, 20).

In order to diagnose OD, the volume-viscosity swallow test (V-VST) is currently recognized as the gold standard (21), however epidemiological studies are often based on the Water Swallow Test (WST) or clinical questionnaires. The use of different assessment methods adds to a wide range of OD prevalence rates in the literature. A cross-country study by Streicher et al. (2017) reported prevalence rates of OD up to 48% using a standardised questionnaire in nursing home residents worldwide (2). Sarabia-Cobo et al. (2016) found a prevalence rate of almost 70% of OD in nursing home residents when using a mixed-method approach including clinical history, physical examination, the EAT-10 (Eating Assessment Tool-10) and the 3 oz - WST (22).

Similar to the diagnosis of OD, a variety of definitions, measurements and tools to determine nutritional status are applied (18) since there is no gold standard or a universal definition for malnutrition in an older population. As a consequence, the literature contains a wide range of prevalence rates of malnutrition among nursing home residents (23). Streicher et al. (2017) reported a prevalence of 16% of malnourished nursing home residents based on anthropometric measurements (2), though prevalence rates of malnutrition up to 38% were found based on the mini nutritional assessment (MNA) in institutionalized older people (24).

Treatments in malnourished residents suffering from OD are of compensative or rehabilitative nature and include e.g. diet modifications, nutritional supplementation, oral-motor therapy, postural techniques and/or facilitation techniques (25). In general, a multidisciplinary approach from an otolaryngologist and/or neurologist and/or gastroenterologist, a clinical geriatrician/ elderly care physician, a radiologist, a speech/ language therapist, a dietician, and a nurse and caregiver, is recommended for safe and efficient swallowing management (26, 27). Due to associated health complications and co-morbidities in older nursing home residents, management and care is complicated, even more so in residents who suffer from dementia (28). Therefore, Dutch nursing homes have comprehensive psychogeriatric or somatic wards, tailored to the needs of the residents (29).

Overall, prevalence rates found for OD and malnutrition are inconclusive and there is some evidence that mortality is even more prevalent in coexisting occurrence of OD and malnutrition (30). However, the association between OD and malnutrition in nursing home residents is still understudied, and especially ward specific literature is lacking. Therefore, this cross-sectional study aimed to delineate associations between OD and malnutrition in Dutch nursing home residents from psychogeriatric and somatic wards.

## Methods

### Study design

Data were obtained from Dutch nursing home residents that participated in the annual cross-sectional National Prevalence Measurement of Quality of Care (LPZ) measurement rounds of 2016 or 2017. The study population included residents of 65 years or older, living in somatic- and psychogeriatric wards of nursing homes across the Netherlands. Data of residents that received palliative care at the day of the measurements were excluded. Detailed information on the study design of the LPZ is available in the study by van Nie-Visser et al. (2013) (31).

### Ethical considerations

Approval for the LPZ was given by the Medical Ethical Committee of Maastricht University and the Academic Hospital Maastricht (Maastricht UMC+, The Netherlands). Participation was voluntary and none of the participating residents, nurses, nursing homes or care institutions received financial compensation.

### Data collection

Data on resident characteristics (age, gender, care dependency and residents’ morbidities), and primary outcome measures (nutritional status, clinically relevant symptoms of oropharyngeal dysphagia and nutritional interventions) were collected on a pre-set measurement date. Trained nurses from different wards within the nursing home collected the data and entered and submitted the data electronically (31).

### Care dependency

The care dependency scale (CDS) is a validated assessment tool to indicate residents’ needs and dependency status. The CDS consists of 15 items, each rated on a five-point Likertscale. A reduced CDS indicated a higher care dependency of the resident (1=highly dependent, 5= almost independent) (32).

### Oropharyngeal dysphagia

The standardized questionnaire of the LPZ was established based on literature and consultation of experts (face validity) and included two questions on clinically relevant symptoms of oropharyngeal dysphagia. Questions asked were: “Does the client have swallowing problems?” (swallowing problems: 0 = no, 1 = yes) and “Does the client sneeze or cough while swallowing food or liquids?” (sneeze/cough while swallowing: 0 = no, 1 = yes).

### Nutritional status: malnutrition

Malnutrition in the nursing home residents was indicated based on the operational definition for malnutrition in older people of the European Society for Clinical Nutrition and Metabolism (ESPEN) (33). Data on anthropometric measurements, weight and height, were collected to determine the Body Mass Index (BMI) for each resident. Residents were considered malnourished with a BMI below 18.5 kg/m^2^, or with a reduced BMI (a BMI below 20 kg/m^2^ in residents aged 65–70 years or a BMI below 22 kg/m^2^ in residents age 70 or older) in combination with recent unintended weight loss (>5% over the past 3 months or >10% indefinite of time).

### Nutritional interventions and referrals

With a multiple-choice question, the nurses could indicate which nutritional interventions the residents received. Nutritional interventions included for example nutritional supplementation and enriched snacks, but also adjustments of food consistency and mealtime-ambiance or referral to a dietician. For residents with symptoms of OD, additional questions on meals and beverage consistencies and referral to a speech-language therapist were incorporated: “Does the client receive mashed meals or thickened beverages because of swallowing problems?” (0 = no, 1 = yes) and “Is the client supervised by a speech-language therapist because of swallowing problems?” (0 = no, 1 = yes).

**Table 1:**
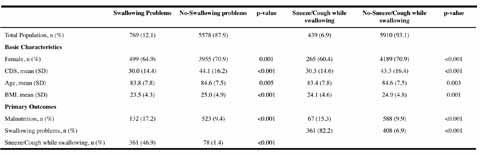
Characteristics and Primary outcomes of residents suffering from OD

### Statistical analysis

Statistical analysis was performed in IBM SPSS statistics 24 (IBM SPSS Statistics, IBM Corporation, Chicago, IL). Normality of the data was determined with QQ-plots. Data of residents with missing values for primary outcomes or outliers (residents with a BMI>70 kg/m^2^ or body height < 108 cm) were eliminated. Of residents that participated in both measurement rounds, 2016 and 2017, only the data of 2017 were included. Prior to analysis, numerical data on BMI and weight loss were recoded into a dichotomous variable on malnutrition based on the ESPEN definition for malnutrition. Independent sample T-tests and Chi-square tests were conducted to check for differences between groups. To assess the association between OD and malnutrition in older nursing home residents, the crude and adjusted prevalence ratios (PR) were subtracted from Cox regression to prevent overestimated associations from logistic regression (34, 35). Confounding factors in the multivariate analysis were based on literature and forward (LR) stepwise regression modeling. The factor ‘measurement round’ was added to the model to control for effect modification. P-values below 0.05 were considered statistically significant.

## Results

### Study population

The study population consisted of 6349 older residents from Dutch nursing homes. Almost two-thirds (66.0%) were residents from the psychogeriatric wards and the remaining residents (34.0%) were admitted to somatic wards. The majority was female (70.2%) with a mean age of 84.5 years (SD 7.5), a mean BMI of 24.8 kg/m^2^ (SD 4.8) and a mean CDS of 42.4 (SD 16.6). Significantly higher mean CDS was found among somatic residents as compared to psychogeriatric residents (p< 0.001). No differences were found between the two study rounds for prevalence rates for malnutrition (2016:10.1% and 2017:10.5%, p=0.584) or for prevalence rates for sneezing/ coughing while swallowing (2016:7.5% and 2017:6.3%, p=0.064). The prevalence of residents with swallowing problems was higher (p=0.017) in 2016 (13.0%) compared to the prevalence of residents with swallowing problems in 2017 (11.1%).

### The prevalence of oropharyngeal dysphagia and malnutrition

Approximately one out of eight residents suffered from swallowing problems (12.1%) and one out of fourteen residents sneezed/coughed while swallowing liquids or solid food(6.9%). If somatic ward residents who suffered from stroke were excluded, the prevalence of residents with swallowing problems was higher (p=0.025) in psychogeriatric wards (11.3%) compared to somatic wards (9.2%). One out of ten residents was malnourished (10.3%) and malnutrition was more often (p = 0.002) seen in psychogeriatric residents (11.1%) compared to somatic residents (8.7%). (Table 1)

Residents with swallowing problems were more often malnourished compared to residents without swallowing problems, with almost one out of every five problematic swallowers being malnourished (17.2%). Almost half of the problematic swallowers indicated additional problematic sneezing/coughing in the act of swallowing (46.9%). Nearly all residents that indicated sneezing/coughing while swallowing had overall problems swallowing (82.2%). (Table 1)

As shown in Table 1, the average CDS was lower (p<0.001), meaning a higher care dependency, in residents with swallowing problems (mean CDS 30.0, SD 14.4) or in residents that sneezed/coughed while swallowing (mean CDS 30.3, SD 14.6) compared to residents without these OD symptoms (respectively mean CDS 44.1, SD 16.2 and mean CDS 43.3, SD 16.4).

**Table 2:**
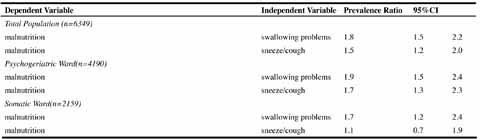
Univariate prevalence ratios from Cox Regression

Among malnourished residents, approximately one out of five was suffering from swallowing problems (20.2%) and one out of ten was sneezing/coughing while swallowing foods or liquid beverages (10.2%).

In comparison to non-malnourished residents (mean CDS 43.0, SD 16.4), the average CDS was lower (P<0.001) in malnourished residents (mean CDS 36.8, SD 34).

**Figure 1:**
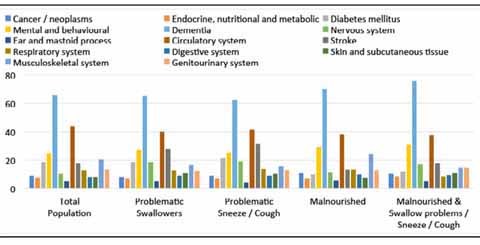
describes the sampling strategy of this study.

### Clinical Diagnosis

More than two-thirds of the residents were diagnosed with dementia (65.6%) and nearly half was diagnosed with disease of the circulatory system (44.1%) (Figure 1). Dementia was also the leading clinical diagnosis among residents with clinically relevant symptoms of OD as swallowing problems and sneezed/ coughed while swallowing. Furthermore, the residents with swallowing problems suffered significantly more often from diseases of the nervous system (excluding paraplegia) (18.7% vs. 9.0%, p < 0.001), stroke (27.7% vs. 16.2%, p < 0.001) and disease of the skin and subcutaneous tissue (10.9% vs. 7.9%, p = 0.004) as compared to residents without swallowing problems. Residents that sneezed/coughed while swallowing were more often diagnosed with diseases of the nervous system too (excluding paraplegia) (19.1% vs. 9.5%, p < 0.001) and stroke (31.4% vs. 16.6%, p < 0.001) as compared to residents that did not sneeze/cough while swallowing.

### Nutritional interventions and referrals

**Figure 2:**
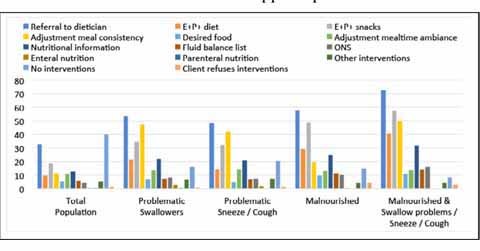
describes the sampling strategy of this study.

Malnourished residents with clinically relevant symptoms of OD were mostly referred to a dietician (57.7%), or received energy (E+) and protein (P+) enriched- diets (29.2%) and/or snacks (48.5%). (Figure 2)

The majority of the residents with clinically relevant symptoms of OD were referred to a speech/language – therapist. A 74.6% of the residents that suffered from swallowing problems and a 79.2% of the residents that were sneezing/coughing while swallowing were referred to a speech/language – therapist.

### Associations - Univariate

Univariate analysis (Table 2) showed an increased risk of malnutrition among nursing home residents suffering from swallowing problems (PR 1.8, 95%CI 1.5–2.2) and among residents that sneezed/coughed while swallowing (PR 1.5, 95%CI 1.2–2.0).

In stratified analysis increased risks for malnutrition amounted to 1.9 (PR 1.9, 95%CI 1.5–2.4) and 1.7 (PR 1.7, 95%CI 1.2–2.4) among residents with swallowing problems at psychogeriatric and somatic wards respectively.

**Table 3:**
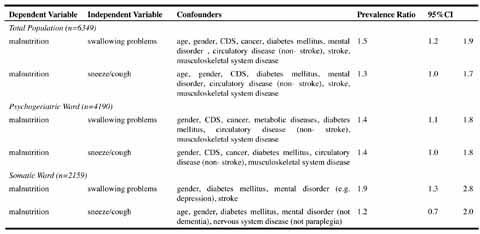
Multivariate prevalence ratios from Cox Regression

Residents at psychogeriatric wards and with sneezing/coughing while swallowing did also show an increased risk of malnutrition (PR 1.7, 95%CI 1.3–2.3).

### Associations - Multivariate

As shown in Table 3, an increased risk of malnutrition was found among residents suffering from swallowing problems (PR 1.5, 95%CI 1.2–1.9).

In stratified analysis, increased risks of malnutrition amounted to 1.4 (PR 1.4, 95%CI 1.1–1.8) and 1.9 (PR 1.9, 95%CI 1.3–2.8) among residents with swallowing problems at psychogeriatric and somatic wards respectively.

## Discussion

This cross sectional prevalence study showed prevalence figures of oropharyngeal dysphagia and malnutrition among older Dutch nursing home residents and revealed significant associations between oropharyngeal dysphagia and malnutrition among these nursing home residents.

The overall prevalence of OD in this current population was lower compared to reported prevalence numbers from previous studies (22, 36). In the current study, the method of diagnosing OD was of an observational clinical nature while in previous studies instrumental visuo-perceptual assessment methods were applied, which are more likely to identify and physiologically interpret the cases of OD (36–38). In addition, cases of OD might have been underreported due to the nurses’ lack of knowledge about how to judge or interpret OD (39), or the residents’ own lack of awareness of their OD (40). They might assume that swallowing difficulties are natural effects of aging (41).

Nevertheless, even without instrumental visuo-perceptual assessment methods, prevalence rates of OD up to 20.2% were found in the current study among malnourished residents. This finding is in line with the results of a similar study by Poisson et al. (2016) among hospitalized older people (36). Almost 20.8% of the patients with a reduced BMI were suffering from OD. In that population, the prevalence of OD was even higher when nutritional status was assessed with the Mini Nutritional Assessment (MNA) (82.1%) or if it was based on serum albumin levels (70.8%). In addition, Poisson et al. (2016) showed that patients with OD had a significantly lower dietary intake compared to patients without OD.

The prevalence of malnutrition in this study population is also relatively low as compared to prevalence rates of malnutrition in the literature (23, 24). However, previous studies were performed in different clinical settings, applied deviant definitions for malnutrition that also included subjects at risk of malnutrition (42) or used methods that tend to overdiagnose malnutrition in this older population (43). In the current study only those who met the ESPEN criteria were considered as malnourished thus nog including those at risk. Furthermore, the problem of malnutrition in frail elderly people has recently received more attention in The Netherlands, which may be a plausible reason for its relatively low prevalence rate.

Interestingly, Poisson et al. (2016) also found an association between malnutrition and oral self-care dependency. Similar results were found in the current study, where lower average care dependency scores (CDS), meaning higher care dependency, were found in residents suffering from OD and in malnourished residents.

With regard to clinically relevant symptoms of OD, subjective swallowing problems were often accompanied by sneezing/coughing in this study. However, some residents without subjective swallowing problems indicated problems with eating due to sneezing/coughing while swallowing, probably also related to dysphagia as it is known that coughing during oral intake is related to penetration or aspiration (44). In the current study, adjusted associations between malnutrition and sneezing/coughing while swallowing were found among psychogeriatric residents. Adjusted associations between malnutrition and swallowing problems were significant among both wards, though more pronounced in somatic wards. Differences between wards can be explained by the group of residents that suffered from a stroke at the somatic wards. According to Foley et al. (45) the chances of malnutrition were more than doubled (OR 2.425, 95%CI 1.264–4.649) among dysphagic residents who had suffered a stroke. Similar results were found in the current study too; residents at the somatic wards had an almost twofold risk for malnutrition (PR 1.9, 95%CI 1.3–2.8) due to swallowing problems.

The group of residents admitted to somatic wards is a relatively small group, approximately one third, of the total population. The majority of residents is admitted to psychogeriatric wards, with dementia as the most frequently occurring clinical diagnosis. A previous study that was conducted in Finnish older nursing home residents revealed two - and three - fold risks for malnutrition due to dementia (OR 2.0, 95%CI 1.5–2.9) and swallowing problems (OR 3.0, 95%CI 2.1–4.4) (46). Swallowing problems may already develop during the early stages of dementia (47) and develop with impaired cognitive-, motor-and sensory mechanisms of swallowing (3).

More specific reference data from the literature on differences between psychogeriatric and somatic wards in nursing homes are lacking at the moment since mainly in Dutch nursing homes these specific distinctions have been made. In addition, to compare the findings of the current study to the literature, take into consideration the difference between varying statistical methodologies to assess associations. Previous studies were based on logistic regression, a commonly used method for the assessment of associations, though known to overestimate associations (34, 35). Therefore, the alternative Cox regression was applied in the current study to assess the association between malnutrition and OD (34, 35).

In the present study nurses reported clinically observed symptoms or complaints of dysphagia. Another method of swallowing assessment may have produced different results although instrumental swallowing assessments such as videofluoroscopy are not available in Dutch nursing homes and fiberoptic endoscopic evaluation of swallowing is not possible on such large scale sample sizes of vulnerable nursing home residents.

No conclusion on causality between OD and malnutrition can be drawn from the current cross-sectional study design, however the clear evidence of an association between OD and malnutrition shows the need for more research on this issue.

## Conclusion

Clinically relevant symptoms of oropharyngeal dysphagia, such as swallowing problems and sneezing/coughing while swallowing are associated with increased risks for malnutrition in psychogeriatric and somatic Dutch nursing home residents. Future research is needed to increase understanding and awareness among affected residents and involved healthcare disciplines to optimize care, tailored to the needs of psychogeriatric and somatic residents with OD and malnutrition in Dutch nursing homes.

*Author Declaration and Ethical Standards:* AvH is employed by Danone Nutricia Research. No other conflict of interests have been declared by the authors. We further confirm that any aspect of the work covered in this manuscript that has involved human patients has been conducted with the ethical approval of all relevant bodies and that such approvals are acknowledged within the manuscript.
